# Slip flow through a non-uniform channel under the influence of transverse magnetic field

**DOI:** 10.1038/s41598-018-31538-8

**Published:** 2018-09-03

**Authors:** Javaria Farooq, Muhammad Mushtaq, Shahzad Munir, M. Ramzan, Jae Dong Chung, Umer Farooq

**Affiliations:** 1 Department of Mathematics, COMSATS Institute of Information Technology, Park road, Tarlai Kalan, 45550 Islamabad, Pakistan; 2 Centre for Automotive Research and Electric Mobility, Universiti Teknologi PETRONAS, Bandar Seri Iskandar, 31750 Tronoh, Perak Malaysia; 3 Department of Computer Science, Bahria University, Islamabad Campus, Islamabad, 44000 Pakistan; 4 Department of Mechanical Engineering, Sejong University, Seoul, 143-747 Korea; 5 Department of Mathematics, Faculty of Science, Jiangsu University, 212013 Zhenjiang, China

## Abstract

This study deals with the steady laminar slip flow of an incompressible Newtonian fluid in a non-uniform permeable channel under the influence of transverse magnetic field. The reabsorption through the wall is accounted for by considering flux as a function of downstream distance. The non-linear coupled partial differential equations of motion are first transformed into a single fourth order partial differential equation and then solved analytically using Adomain decomposition method. Effects of pertinent parameters on different flow properties are discussed by plotting graphs. Results reveal that magnetic field considerably influences the behavior of flow.

## Introduction

The study of flow in ducts with permeable walls is of great practical interest both in engineering as well as bio-physical flows^1–4^. Many processes like membrane filtration, transpiration cooling, blood flow, gaseous diffusion in binary mixtures, renal flow and artificial dialysis are the examples of flows in permeable ducts.

A large number of investigators paid attention towards the experimental and theoretical models of filtration processes. Berman^5^ was the first to study the effect of suction/injection at the permeable wall of the channel. He used similarity solutions along with the perturbation method to analyze the velocity and pressure fields. Yuan *et al*.^6,7^ obtained perturbation solutions for small and large suction Reynolds number by extending the work of Berman^5^. Terrill^8^ found out exact solution for the problem of flow in a porous pipe.

Many authors studied the problem of flow in permeable ducts in context of its application to flow in renal tubule. Macey^9^ determined the solution of a flow problem of viscous fluid through a circular tube by considering linear reabsorption rate at the wall. Kelman^10^ pointed out that the bulk flow rate decays exponentially with the axial distance in renal tubule. Macey^11^ used this condition and observed the parabolic axial velocity profile and found out that mean pressure drop was proportional to the mean axial flow. Marshal and Trowbridge^12^ solved the same problem by making use of physical conditions instead of prescribing flux as a function of downstream distance.

To study the flow problems, authors frequently used the no-slip condition at the solid boundaries. However, this assumption is an idealization and has no empirical justification when fluid flows over a permeable boundary. Many investigators has now accepted that a large class of polymeric materials slip or stick-slip on the solid boundaries. Rao and Rajagopal^13^ studied the flows of a Johnson-Segalman fluid and explained spurt and observed the effects of the slip condition on the flow of Newtonian fluid. Moustafa^14^ illustrated the significance of slip at the wall. It has been justified from literature^15^ that the slip velocity is linearly proportional to the shear rate at the wall. Actually, slip velocity is connected to the thin layer of the fluid that is flowing streamwise just below the permeable wall. The fluid present in this layer is considered to be pulled along by the fluid above the permeable wall. Further, slip is useful in many other applications^16–19^.

Application of magnetohydrodynamics has become quite helpful in various biological problems like in the treatment of different cancer diseases. It is also applicable in engineering problems like electromagnetic casting, plasma confinement and continuous casting process of metals etc. Magnetic field has great influence on the flow of blood. For instance, influence of magnetic field on blood flow is reported by Sinha and Misra^20^. Sud *et al*.^21^ studied the influence of moving magnetic field on the flow of blood. Recently a technology known as nano particles separation technology is studied by many authors. This technology shows that magnetic field can be used to isolate nano particles from plasma with minimum manipulation^22^.

A bulk of literature dealt with the consideration of constant flow rate in permeable ducts. However this is not a good choice for analyzing the flow problems which may have non-uniform normal flow at the walls. Recently authors^23,24^ studied the behavior of physiological flows in various geometries by taking into consideration the variable bulk flow rate due to non-uniform flux at the walls. They used both analytical as well as numerical methods to investigate the effects of different parameters on the flow.

Many researchers^25,26^ worked on blood flow problems in tubes by applying Adomian decomposition method (ADM). This method was developed by Adomian^27^. ADM provides an accurate and computable solutions of the flow problems for sufficiently small number of terms and is proved to be parallel to any supercomputer. The advantage of this method is avoidance of the simplifications which may change the physical behavior of the flow models. It attacks the problems in a straightforward manner without perturbation, linearization and any restrictive assumptions resulting in physically more realistic solutions^27–31^.

In preceding studies, authors considered the channels/tubes of uniform cross-section. But in general, cross-section of renal tubule may vary along its length. Radhakrishnamacharya *et al*.^32^ studied the hydrodynamical aspects of viscous fluid flow in renal tubule by considering it to be a circular tubule of non-uniform cross-section. Chandra and prasad^33^ done the same problem by using starling’s hypothesis. Recently Muthu and Tefshan^34^ studied reabsorption process from the wall of a channel with non-uniform cross-section. Later on Muthu and Teshfa^35^ extended the work^34^ by including the slip effects.

**Figure 1:**
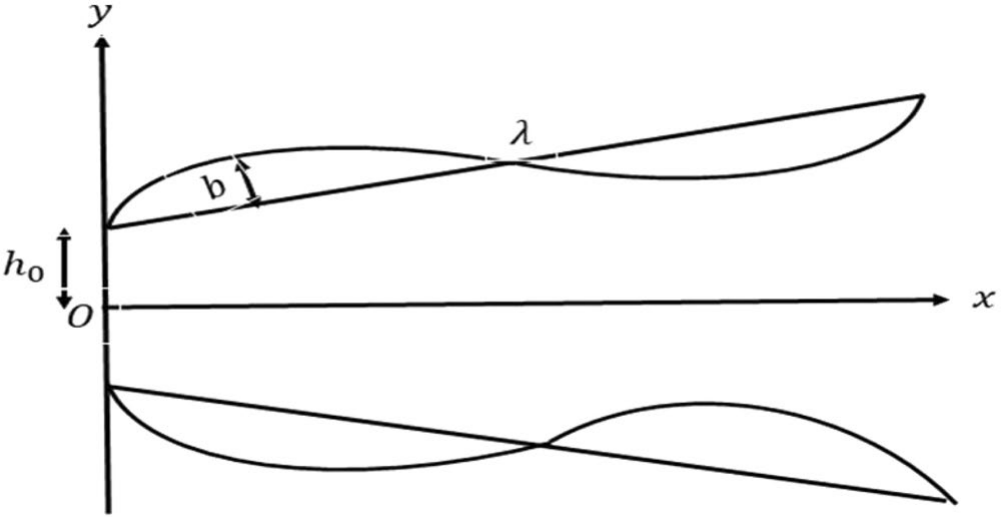
Schematic diagram of flow through a non-uniform channel.

The increasing number of applications of biophysical and industrial flows mentioned above force us to extend already available hydrodynamic solutions to encircle all possible issues and tackle them with appropriate analytical technique. Keeping in view the above studies, the objective of this study is to understand the hydrodynamics of flow of the viscous fluid through a non-uniform channel with slip at permeable wall under the influence of magnetic field. This analysis is carried out by considering flux as a decreasing function of downstream distance. The half-height *h*′(*x*′) of the channel is assumed to vary with axial distance in the following manner

1 $$h^{\prime} (x^{\prime} )={h}_{0}+{m}_{1}x^{\prime} +bsin(\frac{2\pi x^{\prime} }{\lambda }),$$

where *m*_1_ is the slop parameter which depends on the inlet and exit dimensions, *b* is the amplitude, *λ* is the wave length and *h*_0_ is the half height of the channel at *x* = 0 (see Fig. 1).

Full Navier-Stokes equations are solved with non-zero Reynolds number. Influence of reabsorption parameter (*α*), slip coefficient coefficient (*ϕ*) and slop parameter (*m*) on various flow variables in the presence of transverse magnetic field is the main concern of this study. Present work provides a more general form of solution from which already available solutions in literature can be deduced by proper substitutions of pertinent parameters. This study provides a useful information in improving the models for solving different biophysical and engineering problems. Proper knowledge of flow behavior under the influence of magnetic field may be useful in magnetic or electromagnetic therapy as well as in many engineering problems.

## Problem Formulation

Consider a steady flow of an incompressible Newtonian fluid through a permeable channel with slowly varying cross section under the influence of transverse magnetic field. We assume that the induced magnetic field is neglected due to very small magnetic Reynolds number. The half-height of the channel at its inlet is *h*_0_. However it varies along the length of the channel. A rectangular coordinate system (*x*′, *y*′) is chosen, in which is *x*′ taken along the axis of the channel and *y*′ is being normal to it. The volume flow rate is assumed to vary with the downstream distance (see Fig. 1).

The rheological equations of motion governing the flow are given as:

2 $$\frac{\partial u^{\prime} }{\partial x^{\prime} }+\frac{\partial v^{\prime} }{\partial y^{\prime} }=0,$$

3 $$\rho (u^{\prime} \frac{\partial u^{\prime} }{\partial x^{\prime} }+v^{\prime} \frac{\partial u^{\prime} }{\partial y^{\prime} })=-\,\frac{\partial p^{\prime} }{\partial x^{\prime} }+\mu (\frac{{\partial }^{2}u^{\prime} }{\partial x{^{\prime} }^{2}}+\frac{{\partial }^{2}u^{\prime} }{\partial y{^{\prime} }^{2}})-\sigma {B}_{0}^{2}u^{\prime} ,$$

4 $$\rho (u^{\prime} \frac{\partial v^{\prime} }{\partial x^{\prime} }+v^{\prime} \frac{\partial v^{\prime} }{\partial y^{\prime} })=-\,\frac{\partial p^{\prime} }{\partial x^{\prime} }+\mu (\frac{{\partial }^{2}v^{\prime} }{\partial x{^{\prime} }^{2}}+\frac{{\partial }^{2}v^{\prime} }{\partial y{^{\prime} }^{2}}),$$

where *u*′(*x*′, *y*′) and *v*′(*x*′, *y*′) are the axial and transverse components of velocity respectively, *ρ*, *μ* and *p*′(*x*′, *y*′) are the constant density, viscosity and pressure of the fluid respectively *σ* and *B*_0_ are the electrical conductivity and transverse component of magnetic field respectively. The appropriate boundary conditions for the problem under consideration are

Regularity condition:

5 $$\frac{\partial u^{\prime} }{\partial y^{\prime} }=0,\,v^{\prime} =0\,{\rm{at}}\,y^{\prime} =0.$$

Slip at the boundary:

6 $$u^{\prime} +\frac{\partial h^{\prime} }{\partial x^{\prime} }v^{\prime} =-\,\varphi ^{\prime} (\frac{\partial u^{\prime} }{\partial y^{\prime} }+\frac{\partial h^{\prime} }{\partial x^{\prime} }\frac{\partial v^{\prime} }{\partial y^{\prime} })\,{\rm{at}}\,y^{\prime} =h^{\prime} (x^{\prime} ).$$

Bulk flow rate is assumed to be a decreasing function of downstream distance,

7 $$\,Q^{\prime} (x^{\prime} )=2{\int }_{0}^{h^{\prime} (x^{\prime} )}u^{\prime} dy^{\prime} ={Q}_{0}F(\alpha ^{\prime} x^{\prime} ).$$

Pressure at the inlet of the channel is

8 $$\,p^{\prime} (x^{\prime} ,y^{\prime} )=p{^{\prime} }_{0},\,{\rm{at}}\,x^{\prime} =0,\,y^{\prime} =0.$$

In above equations, $\varphi ^{\prime} =\sqrt{\frac{\gamma ^{\prime} }{\beta }}$ is the slip coefficient, in which *γ*′ is the permeability of the wall and *β* is a dimensionless constant which depends on the characteristics of the wall. *F*(*α*′*x*′) = 1 for *α*′ = 0 and decreases with *x*′; *α*′ ≥ 0 is constant reabsorption coefficient; *Q*_0_ is the constant flux across the cross-section at *x*′ = 0 and *h*′(*x*′) is the non-uniform boundary given in Eq. (1).

Introducing the following dimensionless quantities

9 $$\begin{array}{c}x=\frac{x^{\prime} }{\lambda },\,y=\frac{y^{\prime} }{{h}_{0}},\,u=\frac{{h}_{0}}{{Q}_{0}}u^{\prime} ,\,v=\frac{\lambda }{{Q}_{0}}v^{\prime} ,\\ \,\delta =\frac{{h}_{0}}{\lambda },\,\alpha =\alpha ^{\prime} \lambda ,\,h(x)=\frac{h^{\prime} (x^{\prime} )}{{h}_{0}},\end{array}\}$$

where *δ* is the wall variation parameter (ratio of inlet half width to the length of the channel). Eqs (1–3) become

10 $$\frac{\partial u}{\partial x}+\frac{\partial v}{\partial y}=0,$$

11 $$Re\delta (u\frac{\partial u}{\partial x}+v\frac{\partial u}{\partial y})=-\,\frac{\partial p}{\partial x}+{\delta }^{2}\frac{{\partial }^{2}u}{\partial {x}^{2}}+\frac{{\partial }^{2}u}{\partial {y}^{2}}-{H}^{2}u,$$

12 $$Re{\delta }^{3}(u\frac{\partial v}{\partial x}+v\frac{\partial v}{\partial y})=-\,\frac{\partial p}{\partial x}+{\delta }^{2}({\delta }^{2}\frac{{\partial }^{2}v}{\partial {x}^{2}}+\frac{{\partial }^{2}v}{\partial {y}^{2}}),$$

where $Re=\frac{{Q}_{0}}{\upsilon }$ and $H={B}_{0}{h}_{0}\sqrt{\frac{\sigma }{\mu }}$ are the Reynolds number and Hartman number respectively and

$p=\frac{{h}_{0}^{2}}{\mu {Q}_{0}}p^{\prime}$ is the dimensionless pressure.

Boundary conditions (4–7), in dimensionless form are

13 $$\frac{\partial u}{\partial y}=0,\,v=0,\,{\rm{at}}\,y=0,$$

14 $$u+{\delta }^{2}\frac{\partial h}{\partial x}v=-\,\varphi (\frac{\partial u}{\partial y}+{\delta }^{2}\frac{\partial h}{\partial x}\frac{\partial v}{\partial y})\,{\rm{at}}\,y=h(x),$$

15 $$Q(x)=2{\int }_{0}^{h(x)}\,udy=F(\alpha x),$$

16 $$p(x,y)={p}_{0},\,{\rm{at}}\,x=0,\,y=0,$$

where

17 $$\varphi =\frac{\varphi ^{\prime} }{{h}_{0}},\,{\epsilon }=\frac{b}{{h}_{0}},\,m=\frac{{m}_{1}\lambda }{{h}_{0}}.$$

In rectangular coordinates, stream function is defined as

18 $$u=\frac{\partial \psi (x,y)}{\partial y},\,v=\frac{\partial \psi (x,y)}{\partial x}.$$

Making use of Eq. (18) into Eqs (10–12) and eliminating *p* between Eqs (11) and (12), we get the following compatibility equation

19 $${\nabla }^{4}\psi =Re\delta [\frac{\partial \psi }{\partial y}\frac{\partial }{\partial x}({\nabla }^{2}\psi )-\frac{\partial \psi }{\partial x}\frac{\partial }{\partial y}({\nabla }^{2}\psi )]-{H}^{2}\frac{{\partial }^{2}\psi }{\partial {y}^{2}},$$

where

$${\nabla }^{2}={\delta }^{2}\frac{{\partial }^{2}}{\partial {x}^{2}}+\frac{{\partial }^{2}}{\partial {y}^{2}}.$$

Boundary conditions (13–15) take the following form

20 $$\frac{{\partial }^{2}\psi }{\partial {y}^{2}}=0,\,\frac{\partial \psi }{\partial x}=0\,{\rm{at}}\,y=0,$$

21 $$\frac{\partial \psi }{\partial y}-{\delta }^{2}\frac{\partial h}{\partial x}\frac{\partial \psi }{\partial x}=-\,\varphi (\frac{{\partial }^{2}\psi }{\partial {y}^{2}}-{\delta }^{2}\frac{\partial h}{\partial x}\frac{{\partial }^{2}\psi }{\partial x\partial y}),\,{\rm{at}}\,y=h(x),$$

22 $$Q(x)=2{\int }_{0}^{h(x)}\,\frac{\partial \psi }{\partial y}dy=F(\alpha x),$$

We take *F*(*αx*) = *e*^−*αx*^. This assumption is important physiologically as pointed out by Kelman^10^ and used by various authors^11,15,16^.

The problem is now reduced to a fourth order, non-linear partial differential Eq. (19) with non-homogeneous boundary conditions (20–22). Approximate analytical solution of the problem is presented in the next section.

## Solution of the problem

The solution of the problem (19–22) is obtained using Adomian decomposition method as follows^27–31^.

Consider $L=\frac{{\partial }^{2}}{\partial {y}^{2}}$ to be a linear operator. Then Eq. (19) can be rewritten in the following form

23 $${L}^{2}\psi =Re(N\psi )-\frac{{\partial }^{4}\psi }{\partial {x}^{4}}-2L\frac{{\partial }^{2}\psi }{\partial {x}^{2}}+{H}^{2}\frac{{\partial }^{2}\psi }{\partial {y}^{2}},$$

where

24 $$N\psi =\frac{\partial \psi }{\partial y}\frac{\partial }{\partial x}({\nabla }^{2}\psi )-\frac{\partial \psi }{\partial x}\frac{\partial }{\partial y}({\nabla }^{2}\psi ),$$

is the nonlinear term.

Operating *L*^−2^ on both sides of Eq. (23), we get

25 $$\psi ={\psi }_{0}+{L}^{-2}[Re(N\psi )-\frac{{\partial }^{4}\psi }{\partial {x}^{4}}-2L\frac{{\partial }^{2}\psi }{\partial {x}^{2}}+{H}^{2}\frac{{\partial }^{2}\psi }{\partial {y}^{2}}],$$

Remembering that boundary condition terms vanish, operator *L*^−2^ is the two fold pure integral defined as

26 $${L}^{-2}=\iint \iint (.\,)dydydydy,$$

and *ψ*_0_ is the solution of homogeneous equation L*ψ* = 0 which is given below

27 $${\psi }_{0}(x,y)=a(x){y}^{3}+b(x){y}^{2}+c(x)y+d(x)$$

where *a*(*x*), *b*(*x*), *c*(*x*) and *d*(*x*), are constants which are to be determined from boundary conditions.

Decomposing *u*, *Nψ* and *ψ*_0_ as follows^28^

28 $$\psi =\sum _{n=0}^{\infty }\,{\psi }_{n},\,N\psi =\sum _{n=0}^{\infty }\,{A}_{n},\,{\psi }_{0}=\sum _{n=0}^{\infty }\,{\psi }_{0,n},$$

where *A*_*n*_’s are the Adomian polynomials and we have applied double decomposition^28^. Using Eq. (28) into Eq. (25), we arrive at

29 $${\psi }_{n+1}={\psi }_{0,n+1}+{L}^{-2}[Re{A}_{n}-\frac{{\partial }^{4}\psi }{\partial {x}^{4}}-2L\frac{{\partial }^{2}\psi }{\partial {x}^{2}}+{H}^{2}\frac{{\partial }^{2}\psi }{\partial {y}^{2}}],$$

From Eqs (27) and (28), we may write

30 $${y}_{0,n+1}(x,y)={a}_{n}(x){y}^{3}+{b}_{n}(x){y}^{2}+{c}_{n}(x)y+{d}_{n}(x)$$

where the constants are also decomposed as

31 $$a(x)=\sum _{n=0}^{\infty }\,{a}_{n}(x),\,b(x)=\sum _{n=0}^{\infty }\,{b}_{n}(x),\,c(x)=\sum _{n=0}^{\infty }\,{c}_{n}(x),\,d(x)=\sum _{n=0}^{\infty }\,{d}_{n}(x)$$

From Eq. (29), we have

32 $${\psi }_{1}={\psi }_{0,1}+{L}^{-2}[Re{A}_{0}-\frac{{\partial }^{4}\psi }{\partial {x}^{4}}-2L\frac{{\partial }^{2}\psi }{\partial {x}^{2}}+{H}^{2}\frac{{\partial }^{2}\psi }{\partial {y}^{2}}],$$

where

33 $${\psi }_{0,1}(x,y)={a}_{1}(x){y}^{3}+{b}_{1}(x){y}^{2}+{c}_{1}(x)y+{d}_{1}(x)$$

In above equations, Adomian polynomials *A*_0_, *A*_1_, *A*_2_, ... *A*_*n*_ are generated in such way^28^ that

34 $$\begin{array}{c}{A}_{0}\equiv {A}_{0}({\psi }_{0}),\,{A}_{0}\equiv {A}_{0}({\psi }_{0},{\psi }_{1}),\\ \,\,\,\,\,{A}_{2}\equiv {A}_{2}({\psi }_{0,},{\psi }_{1},{\psi }_{2}),\ldots {A}_{n}\equiv {A}_{n}({\psi }_{0,},{\psi }_{1},{\psi }_{2},\ldots {\psi }_{n}).\end{array}$$

Using Eq. (28) into Eq. (24), we can write

35 $${A}_{n}=\frac{\partial {\psi }_{n}}{\partial y}\frac{\partial ({\nabla }^{2}{\psi }_{n})}{\partial x}-\frac{\partial {\psi }_{n}}{\partial x}\frac{\partial ({\nabla }^{2}{\psi }_{n})}{\partial y},$$

From where, we get

36 $${A}_{0}=\frac{\partial {\psi }_{0}}{\partial y}\frac{\partial ({\nabla }^{2}{\psi }_{0})}{\partial x}-\frac{\partial {\psi }_{0}}{\partial x}\frac{\partial ({\nabla }^{2}{\psi }_{0})}{\partial y},$$

Further, boundary conditions (20–22) take the following form

37 $$\begin{array}{c}{\psi }_{0}=0,\,\frac{{\partial }^{2}\,{\psi }_{0}}{\partial {y}^{2}}=0,\,\frac{\partial \,{\psi }_{0}}{\partial x}=0\,{\rm{at}}\,y=0,\\ \frac{\partial \,{\psi }_{0}}{\partial y}-{\delta }^{2}\frac{\partial h}{\partial x}\frac{\partial \,{\psi }_{0}}{\partial x}=-\,\varphi (\frac{{\partial }^{2}\,{\psi }_{0}}{\partial {y}^{2}}-{\delta }^{2}\frac{\partial h}{\partial x}\frac{{\partial }^{2}{\psi }_{0}}{\partial x\partial y}),\,{\rm{at}}\,y=h(x),\end{array}$$

38 $$\begin{array}{c}{\psi }_{1}=0,\,\frac{{\partial }^{2}\,{\psi }_{1}}{\partial {y}^{2}}=0,\,\frac{\partial \,{\psi }_{1}}{\partial x}=0\,{\rm{at}}\,y=0,\\ \frac{\partial {\psi }_{1}}{\partial y}-{\delta }^{2}\frac{\partial h}{\partial x}\frac{\partial {\psi }_{1}}{\partial x}=-\,\varphi (\frac{{\partial }^{2}{\psi }_{1}}{\partial {y}^{2}}-{\delta }^{2}\frac{\partial h}{\partial x}\frac{{\partial }^{2}{\psi }_{1}}{\partial x\partial y}),\,{\rm{at}}\,y=h(x),\end{array}$$

From Eqs (33) and (37), we get *ψ*_0_ as

39 $${\psi }_{0}=f(x){y}^{3}+g(x)y$$

where

40 $$\begin{array}{c}f(x)=\frac{{e}^{-\alpha x}[1+\alpha {\delta }^{2}{h}_{1}(\varphi +h)]}{2{h}^{2}(3\varphi +h+\alpha {\delta }^{2}\varphi h{h}_{1})}\\ g(x)=\frac{{e}^{-\alpha x}[3h(h+2\varphi )+1+\alpha {\delta }^{2}{h}^{2}{h}_{1}(3\varphi +h)]}{2{h}^{2}(3\varphi +h+\alpha {\delta }^{2}\varphi h{h}_{1})}\end{array}$$

*ψ*_1_ can be determined from Eqs (32), (33), (39) and (40) as given below

41 $$\psi 1=a1(x)y3+b1(x)y2+c1(x)y+d1(x)+{\rm{\Omega }}(x){y}^{9}+\eta (x){y}^{7}+\xi (x){y}^{5},$$

where

42 $${\rm{\Omega }}(x)=-\,\frac{Re{\delta }^{2}}{1008}[{f}_{1}{f}_{2}-f{f}_{3}],$$

43 $${\rm{\eta }}(x)=\frac{1}{840}[Re(12f{f}_{1}+{\delta }^{2}(3f{g}_{3}-3{g}_{1}\,{f}_{2}-{f}_{1}{g}_{2}+g{f}_{3}))-{\delta }^{4}{{\rm{f}}}_{4}],$$

44 $$\xi (x)=\frac{1}{120}[Re(g(6{f}_{1}+{\delta }^{2}{g}_{3})-{g}_{1}(6f+{\delta }^{2}{g}_{2}))-{\delta }^{2}(12{f}_{2}+{g}_{4})+6{H}^{2}f].$$

In above equations, subscripts with *f* and *g* denote the order of their derivative with respect to *x*. *a*_1_(*x*), *b*_1_(*x*), *c*_1_(*x*) and *d*_1_(*x*) are obtained from boundary conditions (38) as

45 $$\begin{array}{c}{b}_{1}(x)=0,\,{d}_{1}(x)=0,\\ {a}_{1}(x)=\frac{-{h}^{2}}{3\varphi +h+\alpha {\delta }^{2}\varphi h{h}_{1}^{2}}[\begin{array}{c}3{h}^{2}\eta (7\varphi +h)+2\xi (5\varphi +h)+4{h}^{4}{\rm{\Omega }}(9\varphi +h)\\ +\alpha \varphi {\delta }^{2}h(3{h}^{2}\eta {\eta }_{1}^{2}+2\xi {h}_{1}^{2})+4\alpha \varphi {\delta }^{2}{\rm{\Omega }}{h}_{1}^{2}\end{array}]\\ {c}_{1}(x)=\frac{-1}{3\varphi +h+\alpha {\delta }^{2}\varphi h{h}_{1}^{2}}[\begin{array}{c}2{h}^{6}\eta (9\varphi +h)+{h}^{4}\xi (7\varphi +h)+3{h}^{8}{\rm{\Omega }}(11\varphi +h)\\ +\alpha \varphi {\delta }^{2}{h}^{5}(2{h}^{2}\eta {\eta }_{1}^{2}+\xi {h}_{1}^{2})+3\alpha \varphi {\rm{\Omega }}{h}^{9}{h}_{1}^{2}\end{array}]\end{array}$$

We can obtain the similar expressions for *ψ*_2_, *ψ*_3_ and so on. Since we aimed to find approximate analytical solution therefore, two term approximate solution using Eqs (39–45) can be written as

46 $$\psi ={\rm{\Omega }}{y}^{9}+\eta {y}^{7}+\xi {y}^{5}+m{y}^{3}+ny,$$

where

47 $$\begin{array}{c}m(x)=f(x)+{a}_{1}(x),\\ n(x)=g(x)+{c}_{1}(x)\end{array}$$

Velocity components can readily be obtained by using Eq. (46) into Eq. (18) as

48 $$\begin{array}{c}u=9{\rm{\Omega }}{y}^{8}+7\eta {y}^{6}+5\xi {y}^{4}+3m{y}^{2}+n,\\ v\,=\,-\,{{\rm{\Omega }}}_{1}{y}^{9}+{\eta }_{1}{y}^{7}+{\xi }_{1}{y}^{5}+{m}_{1}{y}^{3}+{n}_{1}y,\end{array}$$

where superscripts with Ω, *η*, *ξ*, *m* and *n* denote their first derivative with respect to *x*.

## Pressure Distribution

Expression for pressure distribution can be obtained by integrating Eq. (11) as

49 $$\begin{array}{c}p(x,y)=\delta \frac{\delta u}{\delta x}+\frac{1}{\delta }\int (\frac{{\delta }^{2}u}{\delta {y}^{2}}-{H}^{2}u)dx-Re(\int u\frac{\delta u}{\delta x}dx+v\frac{\delta u}{\delta y}dx)\\ \,\,\,\,+{\rm{a}}\,{\rm{constant}}\,{\rm{of}}\,{\rm{itegration}}\end{array}$$

Mean presuure drop can be obtained by the formula

50 $$\bar{p}(x)={\int }_{0}^{h(x)}p(x,y)dy.$$

Thus mean pressure drop between *x* = 0 and *x* = *x*_0_ is denoted by

51 $${\rm{\Delta }}\bar{p}({x}_{0})=\bar{p}(0)-\bar{p}({x}_{0}).$$

## Wall shear stress

For two-dimensional flow, wall shear stress in dimensional form is defined as

52 $$\tau {^{\prime} }_{w}=\frac{(\sigma {^{\prime} }_{y^{\prime} y^{\prime} }-\sigma {^{\prime} }_{x^{\prime} x^{\prime} })\frac{dh^{\prime} }{dx^{\prime} }+\sigma {^{\prime} }_{x^{\prime} y^{\prime} }[1-{(\frac{dh^{\prime} }{dx^{\prime} })}^{2}]}{1-{(\frac{dh^{\prime} }{dx^{\prime} })}^{2}}\,at\,y^{\prime} =h^{\prime} (x^{\prime} ),$$

where

53 $$\sigma {^{\prime} }_{x^{\prime} x^{\prime} }=2\mu \frac{\partial u^{\prime} }{\partial x^{\prime} },\,\sigma {^{\prime} }_{y^{\prime} y^{\prime} }=2\mu \frac{\partial v^{\prime} }{\partial x^{\prime} },\,\sigma {^{\prime} }_{x^{\prime} x^{\prime} }=\mu [\frac{\partial u^{\prime} }{\partial y^{\prime} }+\frac{\partial v^{\prime} }{\partial x^{\prime} }].$$

Using Eq. (9), we get shear stress in dimensionless form as

54 $${\tau }_{w}=\frac{2{\delta }^{2}(\frac{\partial v}{\partial y}-\frac{\partial u}{\partial x})\frac{dh}{dx}+(\frac{\partial u}{\partial y}+{\delta }^{2}\frac{\partial v}{\partial x})(1-{\delta }^{2}{(\frac{dh}{dx})}^{2})}{1-{\delta }^{2}{(\frac{dh}{dx})}^{2}}.$$

where

55 $${\tau }_{w}=({h}_{0}^{2}/\mu {Q}_{0})\tau {^{\prime} }_{w}.$$

## Pressure Distribution

Expression for pressure distribution can be obtained by using Eqs (30), (18) and (11) as

56 $$\begin{array}{c}p(x,y)=\delta \frac{\partial u}{\partial x}+\frac{1}{\delta }\int (\frac{{\partial }^{2}u}{\partial {y}^{2}}-{H}^{2}u)dx-Re(\int u\frac{\partial u}{\partial x}dx+\int v\frac{\partial u}{\partial y}dx)\\ \,\,\,\,+{\rm{a}}\,{\rm{constan}}\,{\rm{to}}\,{\rm{fintegration}},\end{array}$$

Mean pressure drop can be obtained by the formula

57 $$\bar{p}(x)={\int }_{0}^{h(x)}p(x,y)dy.$$

Thus, mean pressure drop between *x* = 0 and *x* = *x*_0_ is obtained by

58 $${\rm{\Delta }}\bar{p}({x}_{0})=\bar{p}(0)-\bar{p}({x}_{0}).$$

## Results and Discussion

This analysis is carried out to study the behavior of flow of viscous fluid through a non-uniform permeable channel under the influence of transverse magnetic field. The flux is accounted for by considering as an exponential function of downstream distance. It may be recalled that *δ* characterize the ratio of inlet half-width to the length of the channel, *λ* is the wavelength, *m* is the slope parameter and *α*, *ϕ* and *H* represent the reabsorption, slip and Hartmann number, respectively. The significant characteristics of pertinent parameters on velocity, pressure and shear stress are discussed through graphs. The moderate values of the parameters *ε*(=0.1), *Re*(=1) and *δ*(=0.1) from already existing literature on physiological problems^20,25,35–37^ are chosen in our analysis.

A comparison of present results is made with already published work^35^ in the limiting case of negligible magnetic strength (*H* → 0). For this purpose, numerical values of mean pressure drop over the length (*x*_0_ = 1) of the channel for different values of reabsorption parameter (*α*) and slip parameter (*ϕ*) are computed by software Mathematica^38^ and presented in Table 1. Our results are close to the published results^35^.

**Table 1:** Comparison of pressure drop over the length of the channel for limiting case when *H* → 0 and *Re* = 1, *δ* = 0.1 and *ϕ* = 0.1.

*α*	Published work^35^	Present work
0.5	16.7689	16.7688
1.0	12.9496	12.9493
1.5	10.1895	10.1894
2.0	8.15	8.144711

**Figure 2:**
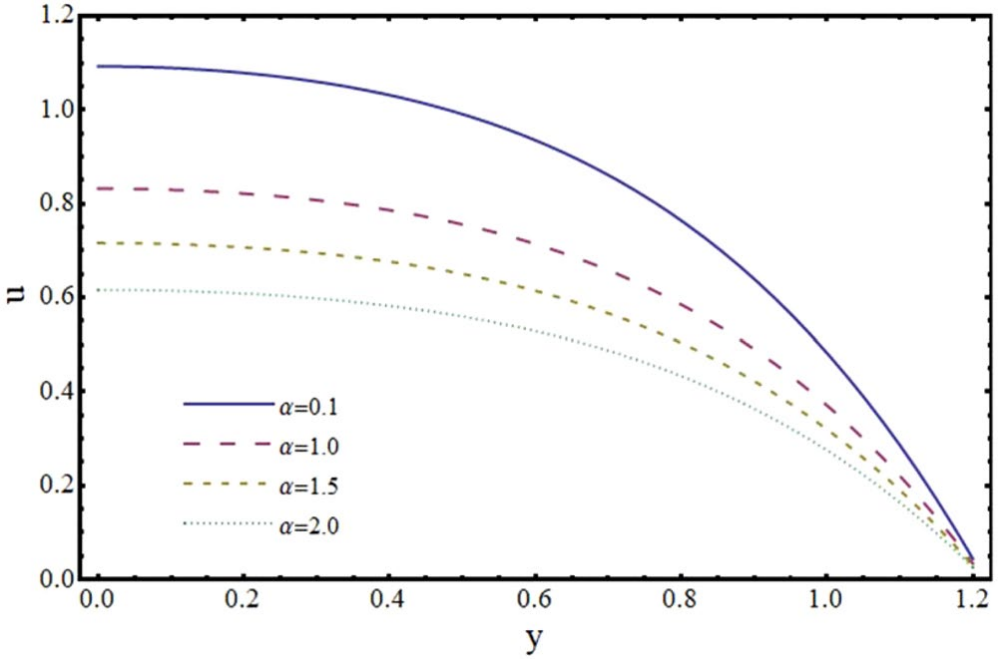
Effect of reabsorption parameter (*α*) on axial velocity (*u*) at *x* = 0.3 when *ϕ* = 0.1, *H* = 2 and *m* = 0.1.

**Figure 3:**
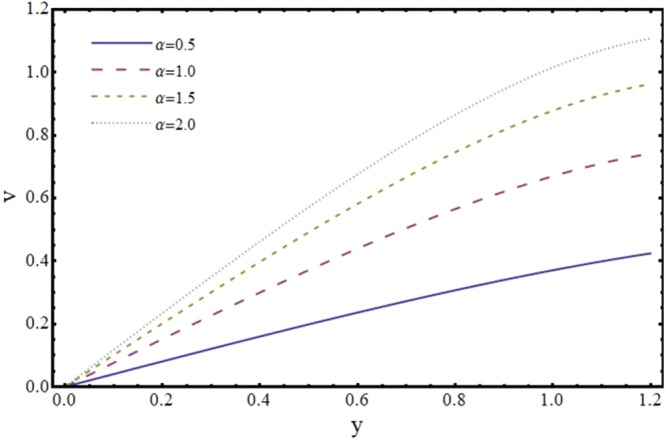
Effect of reabsorption parameter (*α*) on transverse velocity (*v*) at *x* = 0.3 when *ϕ* = 0.1, *H* = 2 and *m* = 0.1.

The effects of various parameters on velocity components (*u*, *v*) and magnitude of wall shear stress (*τ*_*w*_) are discussed by plotting graphs. Figures 2 and 3 portray the impact of reabsorption parameter (*α*) on the axial and transverse velocity profiles. It is witnessed that axial and normal velocity components are diminishing functions of *α*. This is natural because of loss of fluid from walls of the channel and decay of volume flow rate. It is worth mentioning here that the present phenomenon reduces to the case of impermeable walls when *α* → 0.

**Figure 4:**
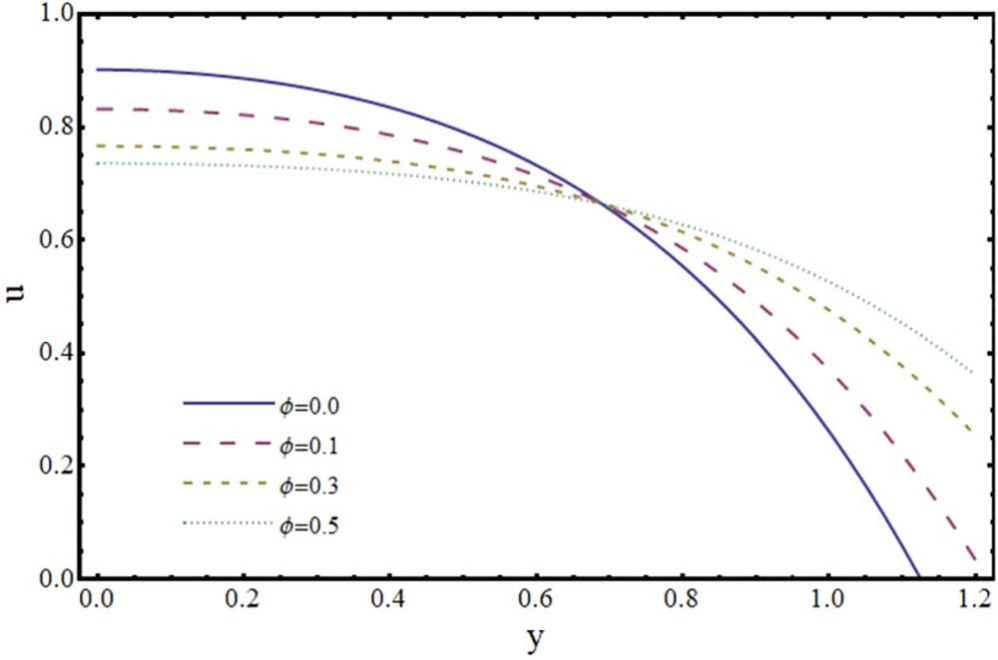
Effect of slip parameter (*ϕ*) on axial velocity (*u*) at *x* = 0.3 when *α* = 1, *H* = 2 and *m* = 0.1.

**Figure 5:**
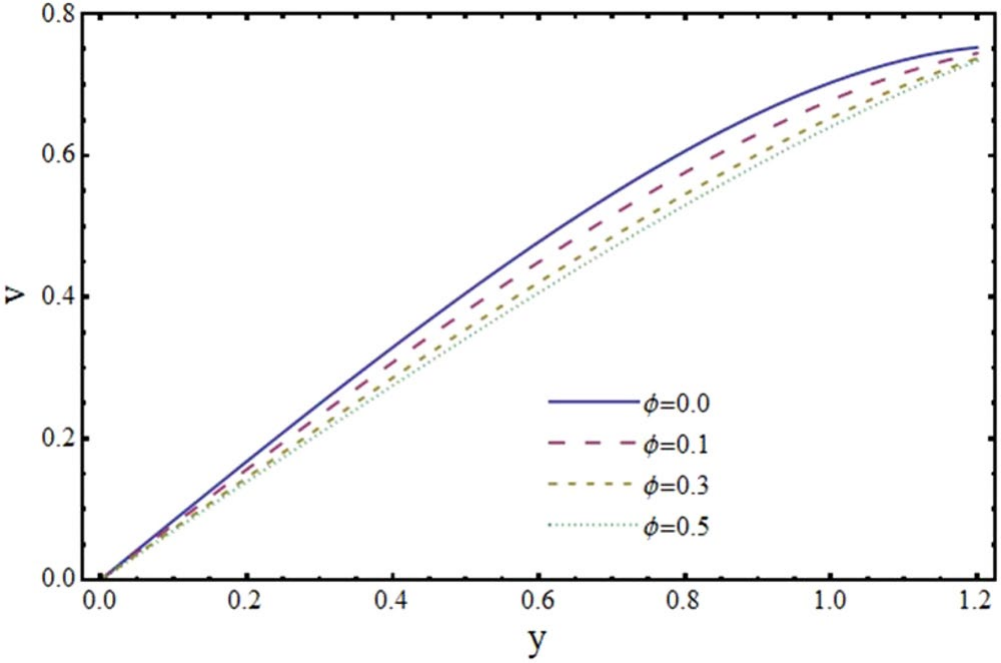
Effect of slip parameter (*ϕ*) on transverse velocity (*v*) at *x* = 0.3 when *α* = 1, *H* = 2 and *m* = 0.1.

The effect of slip parameter *ϕ* on axial and transverse velocity is depicted by Figs 4 and 5. It is noteworthy here that *ϕ* ≠ 0 corresponds to no slip condition and *ϕ* = 0 represents velocity slip at the channel wall. It is noticed that axial velocity *u* shows a decreasing trend near the center and increasing trend near the wall of the channel. This cut off is observed at *y* = 0.6 for particular choice of *ϕ*. This observation is somewhat intriguing and must be discussed by some physical reasoning. As expected axial velocity *u* becomes zero at the wall of the channel for *ϕ* = 0. However there is an increase in *u* for *ϕ* ≠ 0 due to the fact that slip occurs at the wall. Since *u* is proportional to the slip parameter *ϕ* at the wall therefore, by increasing *ϕ*, *u* also increases near the wall. Therefore, for *ϕ* = 0=, *u* goes to zero at the boundary and thus crossover of *u* for *ϕ* = 0.1, *ϕ* = *0*.*3* and *ϕ* = 0.5 is approximately *y* = 0.6. Moreover, Fig. 5 shows that *v* is a decreasing function of *ϕ* As suggested by Kohler^37^, reasonable values of *ϕ* are upto 0.5.

**Figure 6:**
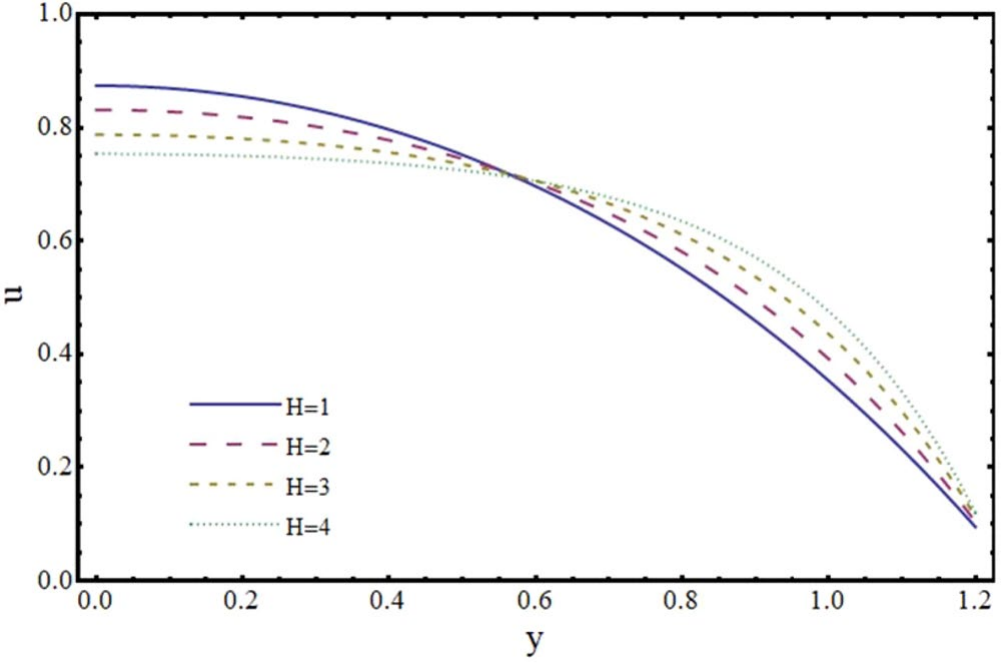
Effect of Hartmann number (*H*) on axial velocity (*u*) at *x* = 0.3 when *α* = 1, *ϕ* = 0.1 and *m* = 0.1.

**Figure 7:**
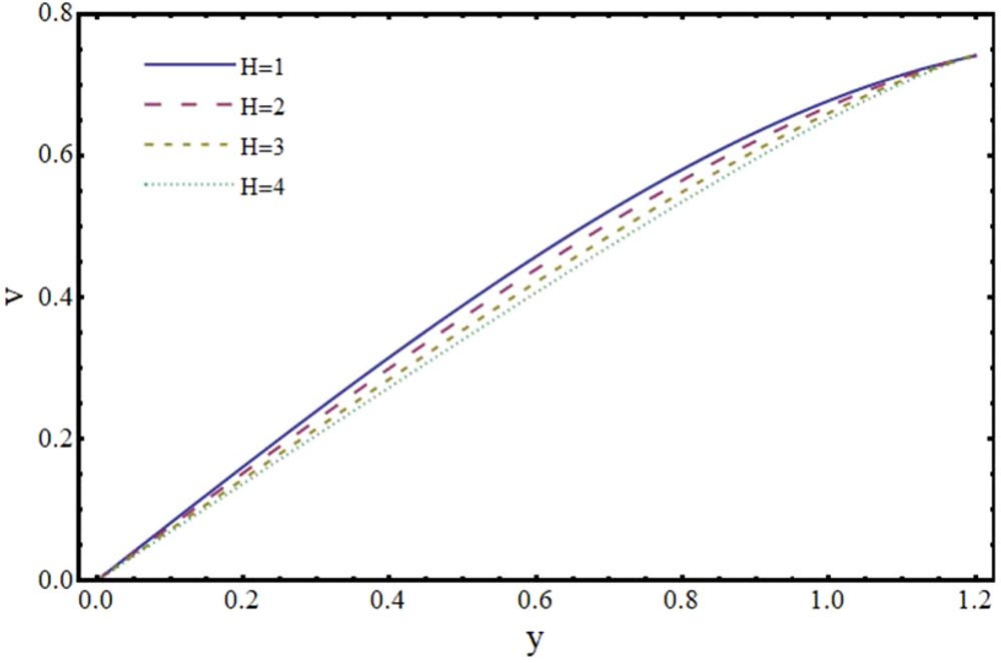
Effect of Hartmann number (*H*) on transverse velocity (*v*) at *x* = 0.3 when *α* = 1, *ϕ* = 0.1 and *m* = 0.1.

**Figure 8:**
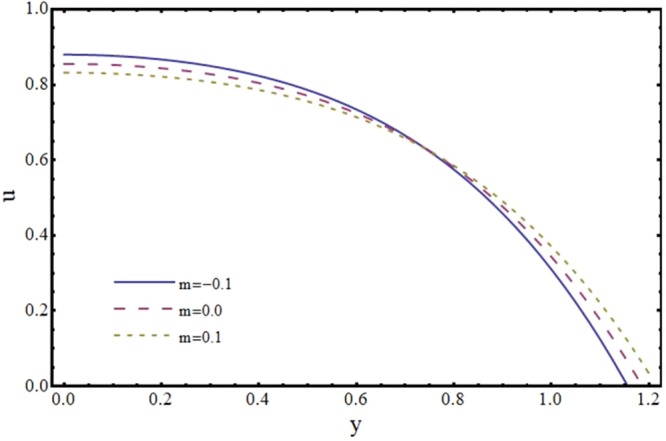
Effect of slope parameter (*m*) on axial velocity (*u*) at *x* = 0.3 when *α* = 1, *ϕ* = 0.1 and *H* = 2.

**Figure 9:**
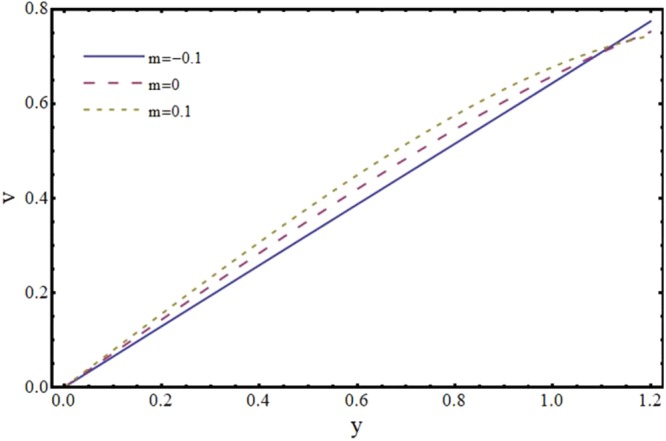
Effect of slope parameter (*m*) on transverse velocity (*v*) at *x* = 0.3 when *α* = 1, *ϕ* = 0.1 and *H* = 2.

**Figure 10:**
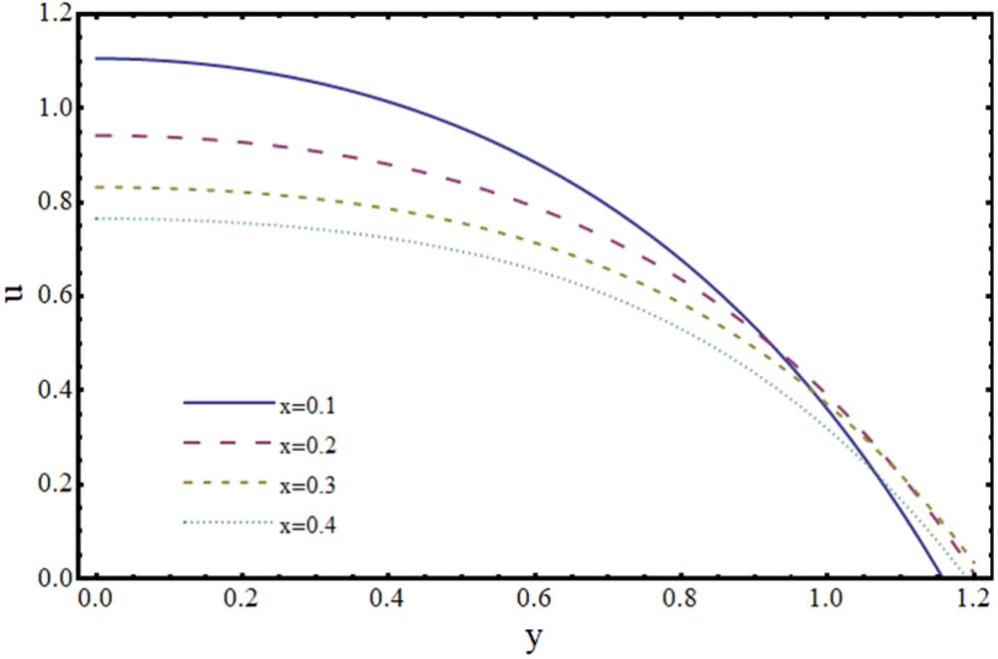
Effect of axial distance (*x*) on axial velocity (*u*) when *α* = 1, *ϕ* = 0.1 and *H* = 2.

**Figure 11:**
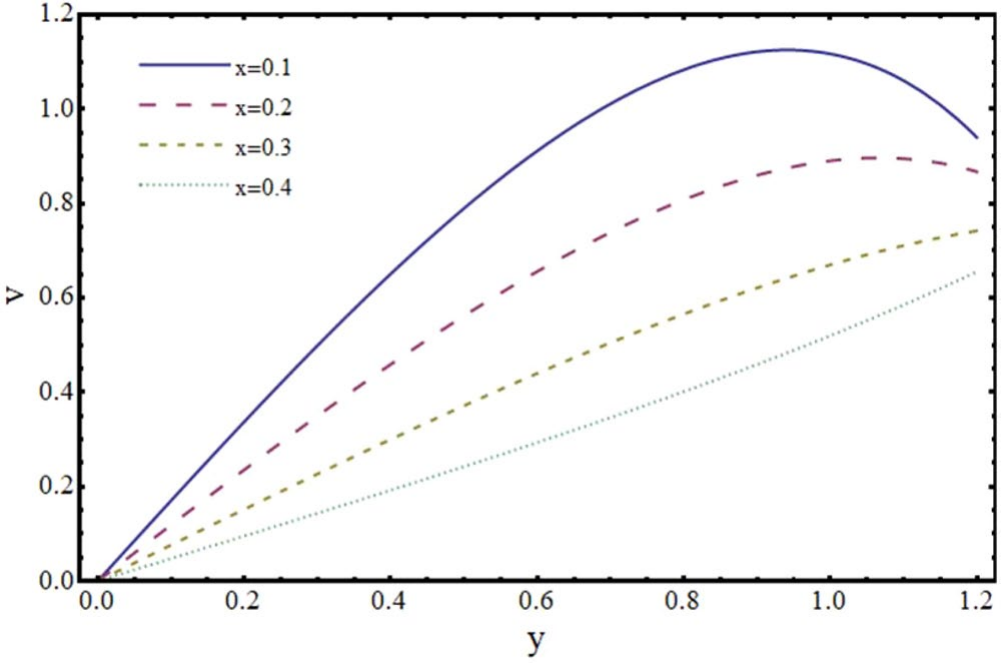
Effect of axial distance (*x*) on transverse velocity (*v*) when *α* = 1, *ϕ* = 0.1 and *H* = 2.

Figures 6 and 7 depict the axial and transverse components of velocity field for different values of Hartmann number (*H*). It is observed from Fig. 6 that *u* decreases upto half of the channel and beyond that it increases. This effect of *H* on *u* near the wall is opposite to the effect of *H* in case of impermeable channel flow where *u* is damped by the application of transverse magnetic field. Physically, rising the parameter *H* produces the Lorentz force. This is a resistive force which suppress the velocity field. This implies that magnetic field strength can be utilized to control the velocity field and hence its application may be important from physiological point of view. It is interesting to note here that present results reduce to the already exist results for *H* = 0^35^. The axial and transverse components of velocity are plotted vs y-axis for different values of slope parameter *m* in Figs 8 and 9. It is observed that *u* has a higher value for divergent channel than a normal or convergent channel near the wall of the channel. Transverse velocity *v* also has higher values for divergent channel than a normal or convergent channel. From Figs 10 and 11, it is noticed that *u* and *v* are diminishing functions of downstream distance. This is due to the fact that reabsorption occurs at the walls of the channel which results in reduction of flux with axial distance.

**Figure 12:**
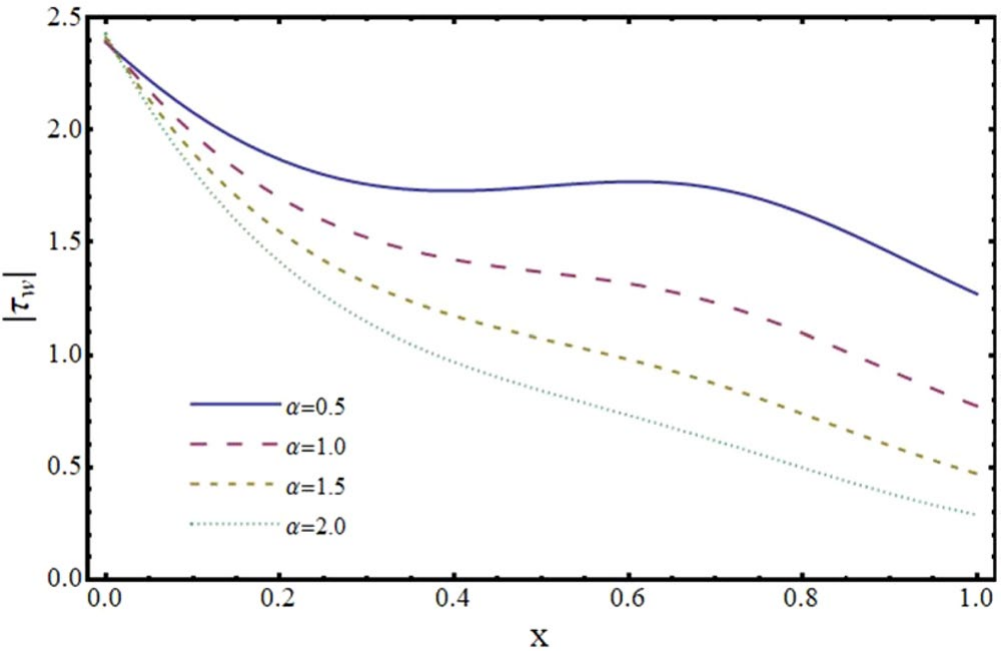
Effect of reabsorption parameter (*α*) on wall shear stress (*τ*_*w*_) when *ϕ* = 0.1, *H* = 2 and *m* = 0.1.

**Figure 13:**
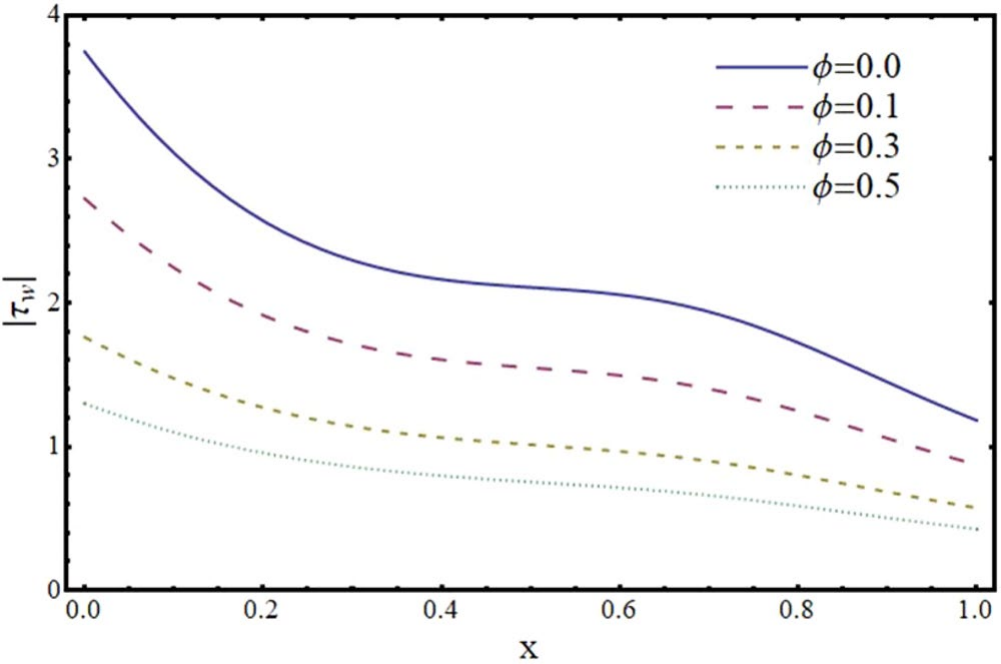
Effect of slip parameter (*ϕ*) on wall shear stress (*τ*_*w*_) when *α* = 1, *H* = 2 and *m* = 0.1.

**Figure 14:**
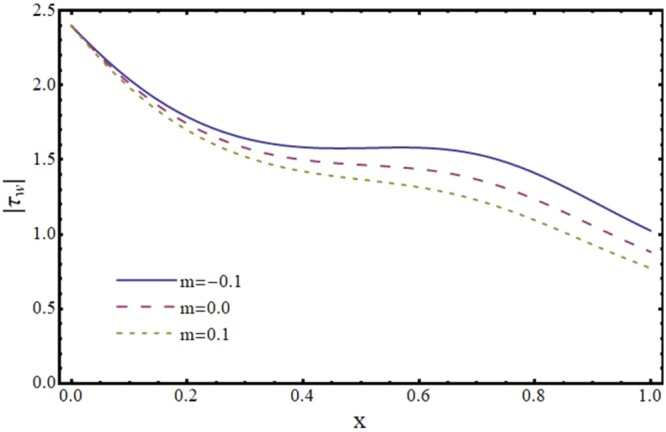
Effect of slope parameter (*m*) on wall shear stress (*τ*_*w*_) when *α* = 1, *ϕ* = 0.1 and *H* = 2.

**Figure 15:**
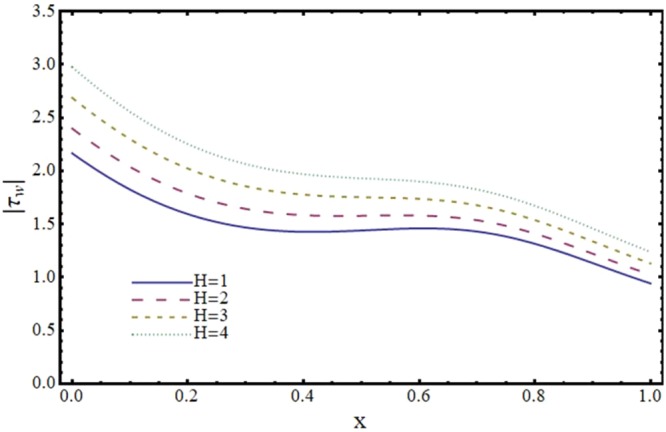
Effect of Hartmann number (*H*) on wall shear stress (*τ*_*w*_) in a convergent channel when *ϕ* = 0.1 and *α* = 1.

**Figure 16:**
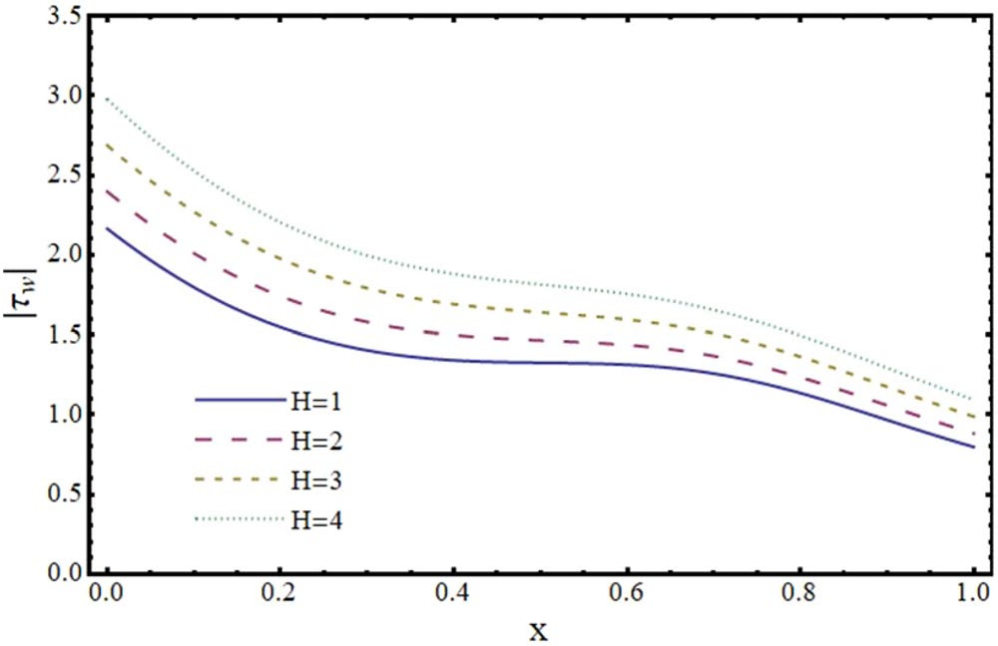
Effect of Hartmann number (*H*) on wall shear stress (*τ*_*w*_) in a normal channel when *ϕ* = 0.1 and *α* = 1.

**Figure 17:**
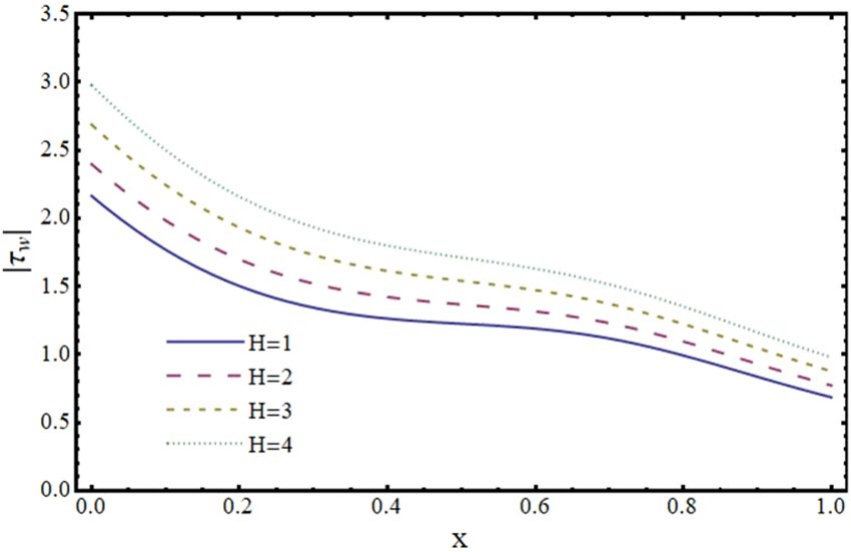
Effect of Hartmann number (*H*) on wall shear stress (*τ*_*w*_) in a divergent channel when *ϕ* = 0.1 and *α* = 1.

Figures 12–17 exhibit the distribution of the magnitude of wall shear stress *τ*_*w*_ with *x* for different values of *α*, *ϕ*, *m* and *H*. The magnitude of *τ*_*w*_ is observed as decreasing function of *α*, *ϕ* and *m* however it is in increasing function of *H*. It is worth mentioning here that *τ*_*w*_ increases by increasing *H* for all the cases of convergent, divergent or normal channel.

## Conclusions

In this paper, the problem of MHD slip flow of a viscous fluid through a non-uniform channel under the influence of transverse magnetic field is discussed. Volume flow rate is assumed to be a function of downstream distance. Following conclusions are made from this study

- The values of *u* and *τ*_*w*_ decrease with *α* while the value of *v* increases with *α*.
- *v* and *τ*_*w*_ decrease by increasing *ϕ*. However, *u* decreases in a region near the center of the channel and inverse is seen near the wall for higher values of *ϕ*.
- By increasing Hartmann number (*H*), *u* and *v* decrease in a region near the center and increase near the wall. Thus velocity field can be controlled by applying appropriate magnetic field.
- *τ*_*w*_ increases by increasing *H*.
- Magnitude of wall shear stress *τ*_*w*_ decreases for a divergent channel in comparison with a convergent channel. *v* and *u* have higher values for a divergent channel.
- All the flow variables decrease with downstream distance. This physically obvious due to loss of fluid from the wall.
- Limiting case of this study i.e., fo r *H* → 0, results are compared with those of Muthu^35^.

Hoping that this study would provide a useful information in improving the already available models for investigating different biophysical and engineering problems. Proper knowledge of flow situations when a magnetic field is being applied on flow in a non-uniform channel may become useful in magnetic or electromagnetic therapy as well as in many engineering problems.
